# Artificial intelligence to predict West Nile virus outbreaks with eco-climatic drivers

**DOI:** 10.1016/j.lanepe.2022.100370

**Published:** 2022-03-30

**Authors:** Zia Farooq, Joacim Rocklöv, Jonas Wallin, Najmeh Abiri, Maquines Odhiambo Sewe, Henrik Sjödin, Jan C. Semenza

**Affiliations:** a Department of public health and clinical medicine, Section of sustainable health, Umeå university, Sweden; b Heidelberg institute of global health and Interdisciplinary center for scientific computing, University of Heidelberg, Im Neuenheimer Feld 205, Heidelberg 69120, Germany; c Department of statistics, Lund university, Sweden

**Keywords:** West Nile virus, *Culex* vectors, Europe, XGBoost, SHAP, Outbreaks management, Early warning systems, forecasting, Climate adaptation, Preparedness, Emerging infectious disease

## Abstract

**Background:**

In Europe, the frequency, intensity, and geographic range of West Nile virus (WNV)-outbreaks have increased over the past decade, with a 7.2-fold increase in 2018 compared to 2017, and a markedly expanded geographic area compared to 2010. The reasons for this increase and range expansion remain largely unknown due to the complexity of the transmission pathways and underlying disease drivers. In a first, we use advanced artificial intelligence to disentangle the contribution of eco-climatic drivers to WNV-outbreaks across Europe using decade-long (2010-2019) data at high spatial resolution.

**Methods:**

We use a high-performance machine learning classifier, XGBoost (eXtreme gradient boosting) combined with state-of-the-art XAI (eXplainable artificial intelligence) methodology to describe the predictive ability and contribution of different drivers of the emergence and transmission of WNV-outbreaks in Europe, respectively.

**Findings:**

Our model, trained on 2010-2017 data achieved an AUC (area under the receiver operating characteristic curve) score of 0.97 and 0.93 when tested with 2018 and 2019 data, respectively, showing a high discriminatory power to classify a WNV-endemic area. Overall, positive summer/spring temperatures anomalies, lower water availability index (NDWI), and drier winter conditions were found to be the main determinants of WNV-outbreaks across Europe. The climate trends of the preceding year in combination with eco-climatic predictors of the first half of the year provided a robust predictive ability of the entire transmission season ahead of time. For the extraordinary 2018 outbreak year, relatively higher spring temperatures and the abundance of *Culex* mosquitoes were the strongest predictors, in addition to past climatic trends.

**Interpretation:**

Our AI-based framework can be deployed to trigger rapid and timely alerts for active surveillance and vector control measures in order to intercept an imminent WNV-outbreak in Europe.

**Funding:**

The work was partially funded by the Swedish Research Council FORMAS for the project ARBOPREVENT (grant agreement 2018-05973).

**Research in context**

**Evidence before this study**

West Nile virus (WNV) is a re-emerging zoonotic pathogen and a threat to both human and animal health. In Europe, there has been a marked range expansion of WNV-outbreaks over the past decades, causing large outbreaks with over 2000 symptomatic cases in 2018 alone. However, the underlying reasons for the WNV re-emergence in Europe remain elusive. We searched PubMed, Web of Science, and Google scholar for primary research articles published between January 2000 and August 2021, to identify eco-climatic drivers and other determinants of WNV outbreaks in Europe. We used combinations of "West Nile", "West Nile virus" in the title and filtered for continental and regional European studies. In a second step we then filtered these articles to included: "risk", "model", "predictor", "estimate", "determinant", "driver" “machine learning” “artificial intelligence”. We stratified the list into eight categories based on class and source of data.

We found that of the regional-level studies only few mentioned eco-climatic drivers at a shorter temporal scale. Moreover, only a few studies applied advanced machine learning algorithms. In effect, no study was identified that used state-of-the-art machine learning and explainable AI frameworks at the European scale that comprehensively elucidates the underlying drivers and determinants at high spatio-temporal resolution of WNV outbreaks.

**Added value of this study**

Eco-climatic drivers and other determinants of WNV, identified in the literature review, were incorporated into a multivariate model at a high spatial resolution, spanning a decade (2010-2019). Using a high-performance machine learning algorithm, we trained a model to discriminate between regions with and without WNV in the time period between 2010-2017. The explainable artificial intelligence (AI) framework could differentiate and rank the most important eco-climatic parameters of WNV outbreaks in Europe on a regional scale. Using test data from 2018 and 2019, this framework performed with high predictive ability to identify regions at risk for WNV-outbreaks. The explainable AI framework could even identify with high accuracy regions at risk during explosive outbreaks, such as 2018, and those at risk during more moderate outbreaks, such as 2019. Most importantly, the AI framework had an ability to even forecast a WNV-outbreak for the entire transmission season ahead of time. We used eco-climatic drivers of the preceding year, and of the first half of the current year, to predict WNV-outbreaks in advance for the latter half of the year.

**Authors should describe here how their findings add value to the existing evidence**

Our study identifies and validates the role of eco-climatic parameters in the emergence and dispersion of WNV in Europe. Our findings point towards the role of climate in the geographic range expansion of WNV-outbreaks in previously naïve regions of Europe. The results of our analysis also underpin the prospect for developing an AI-driven, WNV early-warning system (EWS) based on eco-climatic precursors of WNV. Such an WNV EWS can be deployed to trigger rapid and timely alerts to initiate active surveillance and vector control measures in order to intercept an imminent WNV-outbreak. Operationalizing such an AI-driven EWS for WNV outbreaks in Europe for public health purposes can reduce the disease burden from WNV outbreaks and lessen the associated human and economic costs.

Alt-text: Unlabelled box

## Introduction

West Nile virus (WNV), a member of the family *Flaviviridae*, genus *Flavivirus,* is a re-emerging zoonotic pathogen and a significant threat to both human and animal health. Female mosquitoes of the genus *Culex* are the principal "bridge vectors " in transmitting WNV from birds to humans and equines that serve as incidental and dead-end hosts.^1^ Whilst four-fifths of WNV cases are asymptomatic, the severe cases with neurological manifestations present with seizures, mobility impairment, or loss of consciousness, and can be fatal.^2^

Intermittent WNV-outbreaks have occurred in humans, equines, and avian hosts, with WNV-lineage 2 having overtaken lineage 1 in virulence, in recent years.^3^, ^4^, ^5^, ^6^ In Europe, lineage 1 was replaced by lineage 2 in 2013 and currently dominates the transmission.^6^ Since the emergence of WNV lineage 2 in Europe in 2004,^7^ there has been growing concern about its extensive expansion in several countries including Greece, Romania, and Italy.^8^, ^9^, ^10^

**Figure 1:**
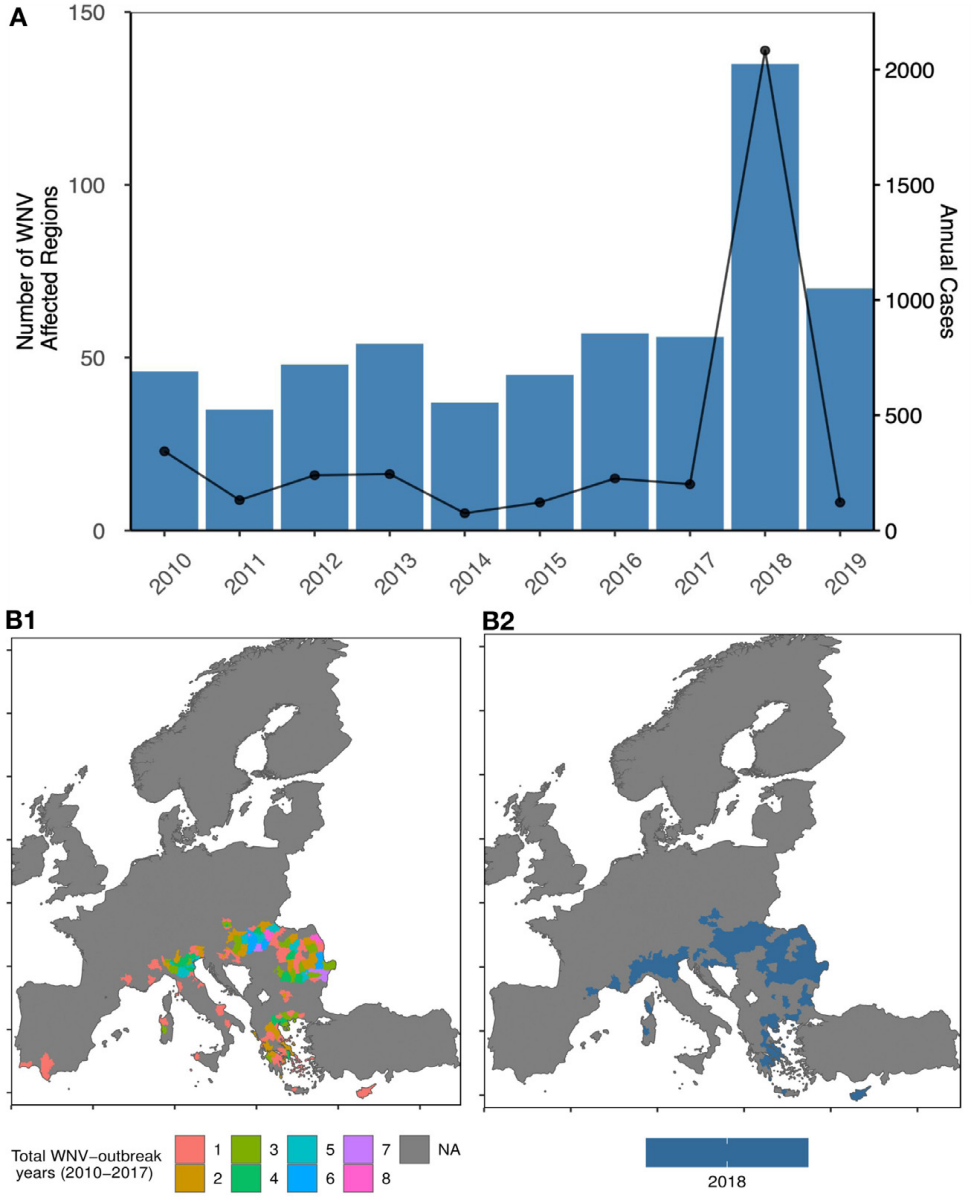
(A) Annual frequency of WNV affected NUTS3 regions and the total human infections cases in Europe, 2010-2019. NUTS3 regions from 28 countries within the EU/EEA were included in this study. Annual count of NUTS3 regions affected by WNV transmission, in Europe (blue bars, left y-axis). The right y-axis shows the annual WNV cases (black curve). (B) Comparison of WNV transmission by NUTS3, 2010-2017 and 2018. B1 represents the cumulative number of years of NUTS3 regions with WNV transmission, 2010-2017, and B2 for 2018.

The geographic occurrence of WNV-outbreaks increased over the past decades, with a 7.2-fold increase in cases in 2018 compared to 2017, and markedly expanded geographic range even when compared to the most extensive outbreak at the time in 2010 (Figure 1).^11^, ^12^, ^13^, ^14^ Specifically, since 2010 ever-expanding areas in Europe have experienced recurrent outbreaks. In 2018, the number of affected NUTS3 (Nomenclature of Territorial Units for Statistics 3) regions increased markedly (Figure 1, B). That year, southern and central Europe witnessed the largest WNV-outbreak ever recorded, with 2083 locally acquired reported human cases and 181 fatalities among them from West Nile neuroinvasive disease (WNND).^13^ The case fatality ratio among these infections was 9%.^13^ Moreover, during the summer months of 2018, greater-than-normal WNV prevalence in mosquito and avian populations were detected.^15^, ^16^, ^17^ In the subsequent year, a higher prevalence of WNV in birds and equines were observed in Germany, prior to discovering WNV cases in humans in 2019.^18^, ^19^, ^20^ For the first time, WNV infection was also detected in a bird in the Netherlands, which foreshadowed the detection of the first human cases in the summer of 2020.^21^

Several abiotic and biotic factors are considered to be the determinants of WNV epidemiology.^22^ Abiotic factors are physical features of the environment, including weather conditions such as temperature, precipitations, landscape features, and land-use, with temperature playing a key role in modulating WNV activity in Europe. The biotic factors are the ones that include host birds of the WNV virus. Bird species population may vary in their susceptibility to WNV infection.^23^ The contribution of factors like climate variability,^24^, ^25^, ^26^, ^27^, ^28^ environmental variables,^25^^,^^26^^,^^29^ WNV vector abundance,^30^^,^^31^ and host migratory birds,^32^^,^^33^ have been examined. Several other factors such as economic conditions,^34^^,^^35^ and sociodemographic characteristics,^36^^,^^37^ are also considered to be important in the WNV epidemiology. A WNV enzootic cycle drives transmission between mosquitoes and birds that act as vectors and amplifying hosts, respectively.^38^ WNV infected humans and equids are the dead-end hosts since they do not contribute to virus transmission. The WNV transmission pathway is very intricate and involves many multifaceted factors; thus, the empirical predictive ability of WNV models is not well refined.

Mechanistically, it is understood that ambient temperature plays a significant role in increasing the vectorial capacity of *Culex* mosquitoes,^39^^,^^40^ thereby accelerating their transmission cycle through the extrinsic incubation period (EIP), the biting rate, and the transmission probability, which in turn results in outbreaks.^18^^,^^39^^,^^41^, ^42^, ^43^ Temperatures as low as 14.0-17.9°*C* have been observed to reduce the EIP of WNV in *Culex pipiens* mosquitoes, necessitating increased transmission levels to trigger infection.^39^^,^^44^ A critical factor in the transmission is the ability of the vector to diapause during the winter.^45^ The above studies draw on experimental results integrated into process-based mathematical conceptual models that can be used to predict transmission dynamics, it is currently less well understood how much each of the climatic, ecological and sociodemographic, vector, and birds factors contribute to the virus transmission when taken together in a modelling framework.

While mechanistic models have been successfully used to reproduce and predict infectious disease transmission, they are often difficult to parameterize and tend to rest, in part, on assumptions of these modelling parameters. In contrast, statistical models fitted to spatio-temporal surveillance data can circumvent such assumptions by estimating all model parameters from data.^25^^,^^46^^,^^47^ These models are favorable due to their lower complexity and higher interpretability of what empirically drives outbreaks.

Recently, high-performance machine learning models have been deployed in infectious disease modelling for this purpose.^48^, ^49^, ^50^, ^51^, ^52^ However, in the context of WNV, only a few regional-level studies with limited explanatory power have employed such methods.^49^^,^^51^^,^^43^ A limitation of these high-performance machine learning models is their high complexity but low interpretability (i.e., black-box type of models) making it difficult to infer public health practice from these models. However, recent advancements in artificial intelligence (AI) have led to the development of explanatory frameworks for uncovering and interpreting the black-box models, often referred to as eXplainable AI (XAI). XAI can thus help us to interpret how highly intricate biological, environmental, and social processes affect disease such as WNV, which, otherwise, would be almost impossible to decipher.^53^

The predictive ability and prospects of developing early warning systems (EWS) to better manage infectious disease outbreaks are of interest to public health practice. In light of current environmental and climatic change, predicting disease outbreaks with eco-climatic drivers is key to preparedness and response. Thus, insights from this analysis, can parameterize EWS with eco-climatic precursors of WNV-outbreaks in Europe and is therefore relevant for the European Climate and Health Observatory, created as part of the European Green Deal,^54^^,^^55^ the EU Adaptation Strategy and EU4Health.^56^^,^^57^ Specifically, the Observatory aims to support Europe in preparing for and adapting to climate change impacts through EWS, information systems, indicators, and tools. This WNV indicator, developed with the help of AI, can support the Observatory in its mandate, as proposed under the European Green Deal.

In this study, we examined the predictive ability of eco-climatic drivers of WNV outbreaks in Europe for the years 2010-2017 using XAI and estimated to what extent the models have the skill to predict the spatio-temporal pattern of the 2018 and 2019 WNV-outbreaks in Europe. As such, this analysis is unique, in its ability to elucidate the underlying drivers of WNV epidemiology in Europe; specifically, it relies on longitudinal predictor data that span a decade. Moreover, a wide range of eco-climatic and other predictors were incorporated into the model, at high spatial resolution (i.e., NUTS3-level) which required high-level computing power and machine learning, to disentangle the determinants of WNV transmission. Advanced AI and XAI methodologies were employed to accomplish these tasks, which has not been attempted before.

We developed a prediction framework using a high-performance machine learning model, XGBoost, for making predictions and the SHAP framework from XAI to rank and uncover the most influential WNV drivers. We then disentangled important drivers of the large 2018-outbreak and further explored the predictive ability of climatic conditions to forecast a WNV-outbreak for the entire transmission season ahead of time. By identifying the key eco-climatic drivers, we lay the foundation for a European-wide AI-based WNV EWS that can help manage climate change induced risk of WNV in Europe.

## Methods

### Data collection

Human WNV cases and eight spatio-temporal predictive feature classes were obtained from the sources described in Table 1. The feature classes with their respective subset of features are also listed in Table 1. Specifically, annual human WNV case count records were obtained from the *European Center for Disease Prevention and Control* (ECDC), aggregated by NUTS3 regions.^58^ Symptomatic human WNV cases, including neuroinvasive WNV disease, were included in the analysis. Infections with unknown aetiology or year of diagnosis were excluded.

**Table 1:** Spatio-temporal explanatory and response features for the training and test data sets of the WNV model, Europe, 2010-2019.

Spatial Range	Temporal Span	Feature Class	Features category / Data Level (unit)	Features names as in model (= Features Description)
EU/EEA NUTS3	2010-2019	Climate ^59^	Temperature / quarter (°C)	*min_temp_*i* = Minimum temperature *mean_temp_*i* = Mean temperature *max_temp_*i* = Maximum temperature
			Precipitation / quarter (mm)	*prec_*i* = Total precipitation per quarter
		Bioclimatic ^59^^,^^60^	Temperature-related bioclimatic features bio1-bio11/ year (°*C*)	bio1 = Mean annual temperature bio2 = Mean diurnal range bio3= Isothermality -(bio2/bio7) x 100 bio4 = Temperature seasonality^+^ bio5 = Maximum temperature of warmest month bio6= Minimum temperature of coldest month bio7 =Temperature annual range (bio5- bio6) bio8= Mean temperature of the wettest quarter bio9 = Mean temperature of driest quarter bio10 = Mean temperature of warmest quarter bio11 = Mean temperature of coldest quarter
			Precipitation-related bioclimatic features bio12-bio19/ year (mm)	bio12 = Total annual precipitation bio13 = Precipitation of wettest month bio14 = Precipitation of driest month bio15 = Precipitation seasonality bio16 = Precipitation of wettest quarter bio17 = Precipitation of driest quarter bio18 = Precipitation of warmest quarter bio19 = Precipitation of coldest quarter
		Environmental^59^^,^^61^	Vegetation index/quarter ([-1,1])	*ndvi_*i* = Normalized difference vegetation index
			Water availability Index/ quarter ([-1,1])	*mndwi_q*i* = Normalized difference water index
		Demographic ^63^	Male age -structured Population / year	M_TOTAL=Total Males, M_Y_LT15=Males <15 years M_Y15-64 = Males 15-64 years M_Y_GE65= Males ≥ 65 years
			Female age-structured population / year	F_TOTAL = Total Females F_Y_LT15 = Female s<15 years F_Y15-64 = Females 15-64 years F_Y_GE65 = Females ≥ 65 years
		Economic ^63^	Income / year (Million Euros)	MIO_EUR = Annual mean regional income
		Trade ^63^	Goods trading /year (Tons)	l_TOTAL = Annual trading loading
				unl_TOTAL = Annual trading unloading
		Vectors ^58^	*Culex pipiens* / year	dist_Culex.pipiens = abundance of Culex pipiens
			*Culex modestus* / year	dist_Culex.modestus= abundance of Culex modestus
		Birds ^64^	*Passeriformes* order / year	local birds of *Passeriformes* order **
		WNV cases ^58^	Response feature (binary)	wnv_case = NUTS3 region with (1) and without (0) reported human WNV cases

Note: *In these feature names, ‘i’ has is replaced by 01,02,03,04, representing the respective quarter of the year

** Please see ref^64^ for details.

+ The amount of temperature variation over a given period is based on the ratio of the standard deviation of the monthly mean temperatures to the mean monthly temperature.^69^

The climate data were acquired from the *Copernicus Climate Change Service* (C3S) database in Europe.^59^ All climatic variables were averaged into four quarters. To provide covariates for the complex interactions between WNV vector species and climate, a set of 19 bioclimatic features (*bio1-bio19*) were derived from temperature and precipitation data.^60^ The quarterly data of environmental features, Normalized Difference Water Index (NDWI), and Normalized Difference Vegetation Index (NDVI) were included in the analysis. NDWI data were derived from Google Earth Engine using the R package '*rgee'*.^61^ The NDVI data were derived from the *C3S* database.^59^ Since both *Culex pipiens* and *Culex modestus* species are competent vectors of WNV and are well established in southern Europe,^62^ their yearly presence and abundance data were obtained from ECDC.^58^ To assess WNV notification associated with population demographic, age-and sex-stratified data were also incorporated as potential covariates. Similarly, annual regional income data were included to assess the association of socio-economic status with WNV transmission by region. The annual trading data was also used as potential covariates. All these features were extracted from R's *Eurostat* package*.*^63^ The local *passeriform* host birds data obtained from *European Environment Agency* (EEA),^64^ were also incorporated at the regional level.

### Model selection

After the geocoded spatio-temporal data of explanatory features were prepared for each NUTS3 region, the final data set was split into training and test sets. The 2010-2017 data were used for training the model while 2018 and 2019 data were chosen for testing. The influence of the determinants was presented by dividing regions into two groups based on transmission activity in a specific year. Then, the supervised machine learning task used a model to learn highly complex features interactions in the data during the training period to classify whether a region had a WNV presence. Once trained, the model would then predict the WNV-outbreaks of the year 2018 and 2019, respectively. The goal was to assess the model's predictive ability for both contained (2019) and more expansive (2018) outbreaks.

We used the XGBoost machine learning algorithm, a high-performance gradient boosting ensemble of decision trees widely used for classification and regression tasks.^65^ The algorithm uses splits, i.e., it iteratively selects the features that best separate the data into two groups. Requiring the least data preprocessing and feature engineering and many tuning hyperparameters to optimize make XGBoost an ideal candidate for highly complex, nonlinear, sparse, and imbalanced classification data such as ours (Appendix; Machine learning algorithm and model selection).

However, the results of XGBoost are often not straightforward to interpret. Also, its internal feature importance metrics cannot quantify a single observation-level feature influence on the overall model predictions. We, therefore, post-processed the model results with SHAP (SHaply Additive Explanation), a game-theoretic,^66^ XAI framework, developed recently.^67^ SHAP ranks feature importance by comparing what a model predicts with and without the feature for all possible combinations of features at every single observation. The features are then ranked according to their contribution for each observation and averaged across observations. The SHAP method enables us to identify drivers of WNV transmission by the NUTS3 region.^53^^,^^68^

Furthermore, to inspect the impact of the preceding year's climate trends and to make within-year WNV-outbreak predictions, we applied and analyzed the XGBoost to four separate data sets (Q1-Q4). In the following text, XGBoost combined with these data sets are called model-Q1, model-Q2, etc. These models differed concerning their feature space and the data selection process. Specifically, for the model-Q1, we used only the first quarter (January-March) data of a year for the climate and environmental features. For features classes with yearly data (see Table 1), the preceding year's data was assigned to their corresponding features. The bioclimatic features in the model represented the recent past (or preceding year's climate trends).

Similarly, for model-Q2, the data for the quarterly feature classes consisted of both the first and second quarters (January-June) features of the same year and the previous years for the rest of the features. A similar process was repeated for the model-Q3. Ultimately, for model-Q4, all the same year's data with the entire feature space was considered in the model.

### Cross-validation and hyperparameter tuning

Machine learning models can be trained with different training and validation strategies like hold-out, k-fold cross-validation (CV), and nested cross-validation. We opted for a more computationally expensive yet robust k-fold cross-validation approach to avoid any overfitting/underfitting and to determine if the model generalizes well to the data. This was done for the training data sets of each model using a 5-fold cross-validation approach. The XGBoost randomly partitions the training data into k-folds (subsets) of equal size. The performance of a model is evaluated from the average score it achieves on each of the created folds.

The performance of machine learning algorithms can be sensitive to their hyperparameters. The tree-based XGBoost comes with a variety of hyperparameters, and the model performance can be improved by tuning and optimizing these parameters. While few parameters are general and depend on the nature of machine learning, others control the performance of the booster algorithm.^65^ Some of these hyperparameters of importance while doing cross-validation are *nrounds* – which represent the number of trees to grow for the classification tasks and should be tuned. While *eta* controls the model's learning rate from the data patterns, *gamma* controls the regularization part of the cost-function and is critical in preventing the model overfitting. The hyperparameter *min_child_weight is* important to block any potential feature interactions causing overfitting. Another crucial parameter for the imbalanced data sets is the *scale_pos_weight-* the ratio of positive classes to the negative classes to handle the class imbalance (Appendix; Imputation of the incomplete dataset using XGBoost). Similarly, *lambda* is tuned during cross-validation and controls the *L2* regularization on weights. The hyperparameter *max_depth,* controls the depth of the tree and is important to tune. Lastly, *max_delta_step* helps in binary classification problems with extreme class imbalance*.*^65^

In our analyses, these hyperparameters were tuned for every model separately during the cross-validation process by creating a random search for the best combination of parameters. The performance of all models was evaluated and compared using the *logloss* score both for cross-fold training and validation sets as well as for out-of-sample test data sets. *Logloss* represents how close the prediction probability is to the corresponding actual value (0 or 1, here). The closer the predicted probability to the actual value, the lower is the *logloss* score. The tuned set of hyperparameters of each of the four models is listed in Table A2 of the Appendix.

Besides *logloss*, models were evaluated using an AUC score to assess how well they distinguish between two classes irrespective of the classification threshold (Appendix; Table A3). Additionally, various threshold-dependent classification metrics were also computed and compared at thresholds 0.1,0.2, and 0.5 (Appendix; Table A3). A comprehensive analysis for the data set with missing observations was also performed (Appendix; Imputation of the incomplete dataset using XGBoost).

### Role of the funding source

The funder had no influence in the study design, data collection, data analysis, interpretations, and writing of the report.

## Results

### Selecting the best model

**Figure 2:**
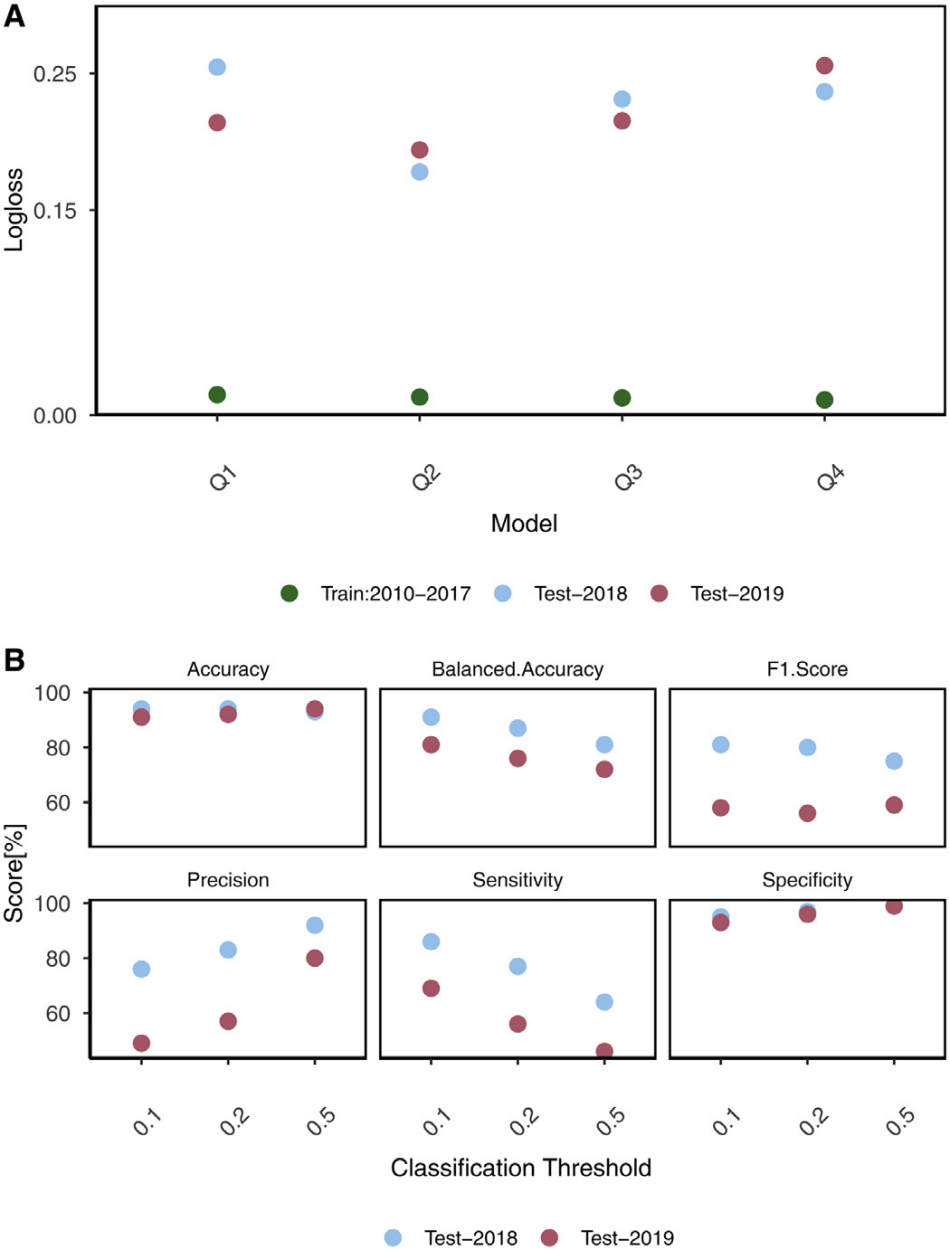
(A) *Logloss* score of all models by quarters (Q1-Q4) of the year, Europe 2010-2019. The *logloss* metrics of all the models are shown for the training data (green circle) and both test data sets, i.e., the year 2018 (light blue) and 2019 (red) of the WNV-outbreaks in Europe. The model-Q2, had the minimum *logloss* score for both test data sets, hence the best model. (B) Performance metrics of model-Q2 on test data sets. For three classification thresholds shown on the x-axis, the accuracy, balanced accuracy, and specificity score remained more consistent for the test data sets than the other three, i.e., F1 score, precision, and sensitivity.

We evaluated the performance of all the models using the XGBoost algorithm using *logloss* for model selection. While all the models performed identically on training data, results showed that model-Q2 performed best on both test data sets (Figure 2, A). The *logloss* score for model-Q2, i.e., the model with climate conditions up to quarter 2 from the same year, remained more consistent for both the test sets. The AUC score - the probability that a randomly selected positive outbreak event will be assigned a higher probability - indicates how well the model discriminates between positive (1) and negative (0) classes irrespective of the classification threshold. These scores for the 2018 and 2019 test data were 0.97 and 0.93, respectively, showing the model's high capability of discriminating between two classes.

For different classification thresholds, various performance metrics of the model-Q2 were also estimated on both test sets. For 2018, the model achieved 86% sensitivity and 95% specificity at a 0.1 classification threshold (Figure 2, B). For 2019, the metrics were 69% and 93%, respectively. Thus, model performance was optimized for an outbreak year, which has public health implications. Both the test data sets had a high-class imbalance in favor of negative classes (0). For 2018, 85% of observations were regions with no prior WNV presence (0) compared to 15% with WNV presence (1), whereas the percentage further dropped for 2019; 91% compared to 9%. Various performance metrics of all the models at three different classification thresholds (0.1, 0.2, 0.5) were also estimated (Appendix; Table A3).

### Key drivers of WNV-outbreaks across Europe

**Figure 3:**
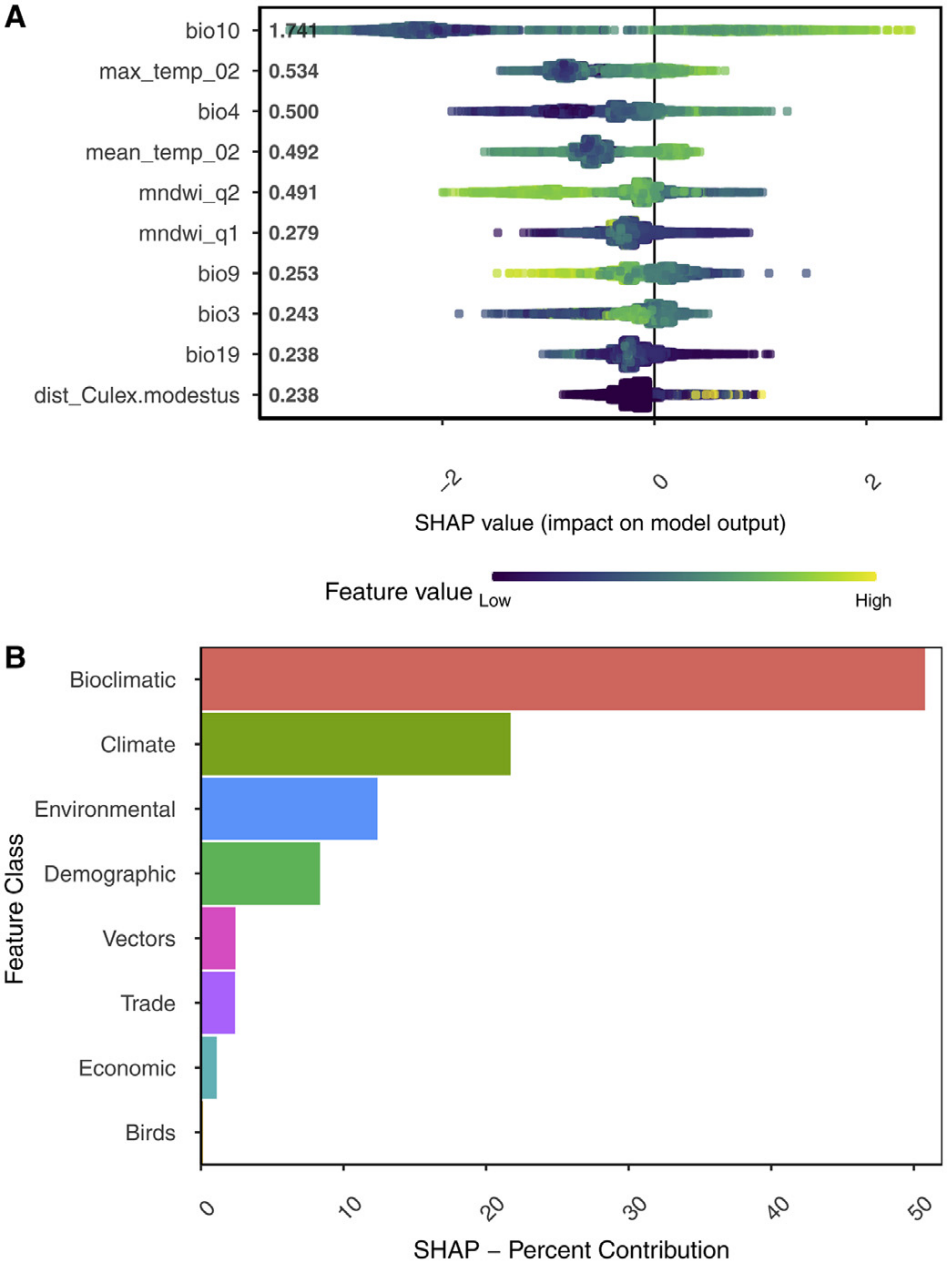
(A) Summary plot: Top-10 most important SHAP predicted features of the WNV model, Europe 2010-2019. The y-axis indicates the variable name in order of importance from top to bottom. The value next to each is the mean absolute SHAP value, the higher the value, the more important the feature. The x-axis represents the SHAP values showing the change in *log-odds*. Gradient color indicates the original values feature. Each point corresponds to a feature value of the original data. The most noteworthy feature is the *bio10*-the mean temperature of the warmest quarter from the preceding year, followed by the maximum temperature of the 2nd quarter of the same year (*max_temp_02*) and the temperature seasonality *(bio4)* of the preceding year. (B) Ranking of the feature classes. Summation and ranking of each feature class from the training period estimated from the European-wide SHAP score of each feature. The horizontal axis represents the SHAP estimated aggregation of features per class. The vertical axis represents the feature class.

We quantified and ranked the key drivers of WNV-outbreaks using SHAP to quantify the European-wide predictions. The most influential feature was the mean temperature of the warmest quarter (*bio10*), followed by the maximum temperature of the 2nd quarter *(max_temp_02)* and temperature seasonality (*bio4)* (Figure 3, A). Interestingly, the NDWI of the second and first quarters were ranked at 5^th^ and 6^th^, respectively, in importance by SHAP. It was found that the regions with low NDWI during the first half of the year were at a higher risk of WNV-outbreaks. Other influential features were the isothermality *(bio3)*, the precipitation of the coldest quarter *(bio19),* and the abundance of *Culex modestus* mosquitoes.

### Preceding year's climate trends – key determinants of WNV-outbreaks

Here, we explored the percent contribution of features classes based on SHAP's European-wide score of each feature. This score was first converted as percent contribution per feature and subsequently aggregated according to the feature class. The 'bioclimate' feature class representing the preceding year's climate conditions alone makes 50% of the total contributions (Figure 3, B). It is followed by 'climate' and 'environmental' features classes that collectively make 35% of overall contributions. They are followed by the rest of the feature classes making approximately 15% contributions collectively.

### Identifying the drivers of the extraordinary 2018 WNV-outbreak

**Figure 4:**
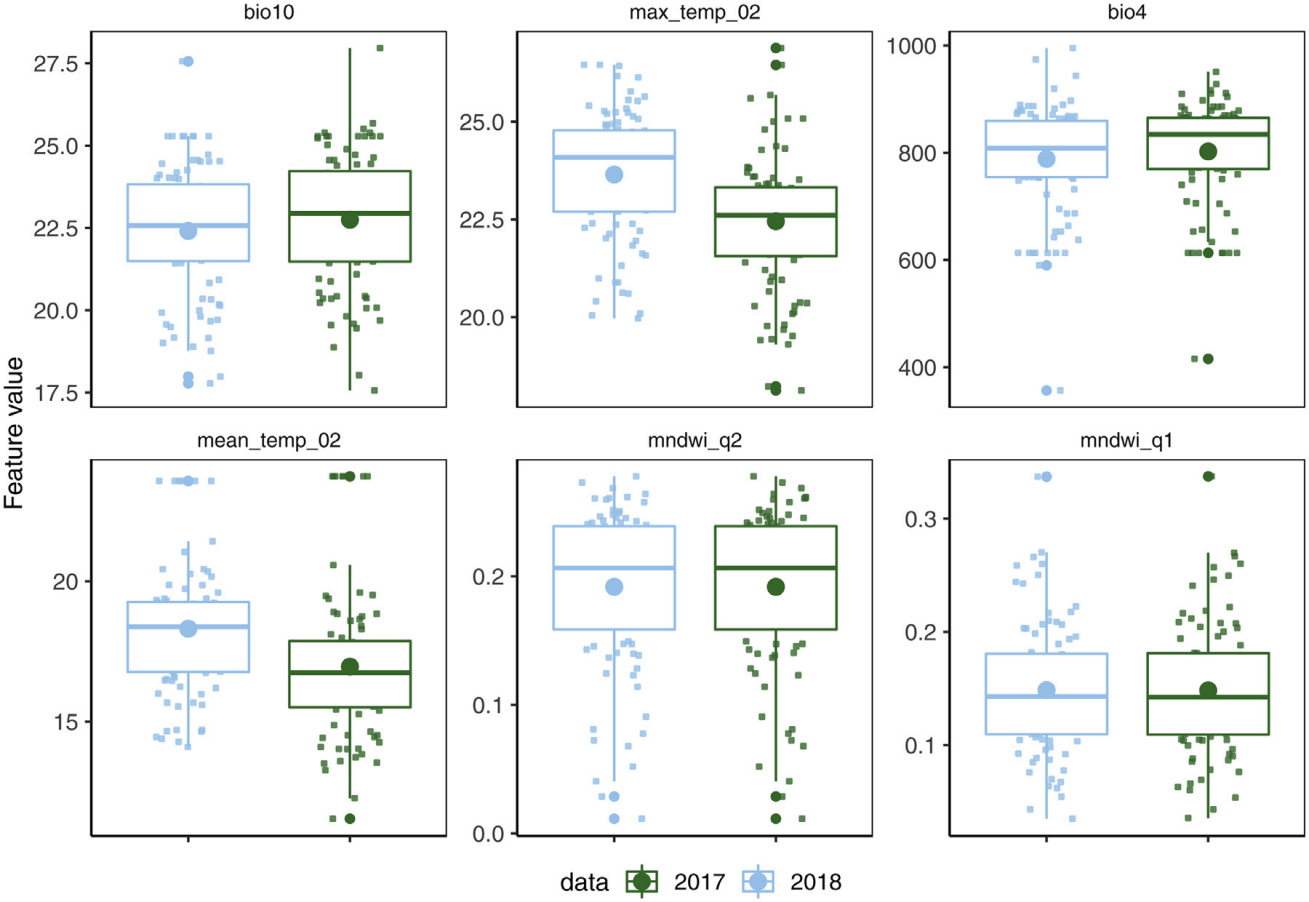
Comparison of observed data of top SHAP predicted features. The boxplot and the mean value (large solid circle) of top-6 most influential features predicted by SHAP for the NUTS regions with a WNV presence in 2018 but not in the preceding year. The regions are compared using the values of the observed features during 2017 (green color) with the 2018 data (light blue).

The model results explored and differentiated the extraordinary outbreak in 2018 compared to the other annual outbreaks. We identified 88 NUTS3 regions with WNV transmission in 2018 but not in the preceding year. The observed features values of the top-6 most influential SHAP predicted features class-wise from the year 2017 and 2018 data sets were analyzed for these regions (Figure 4, A). It was found that the mean temperature of the warmest quarter (*bio10*) during the year 2018 did not differ significantly (p> o.o5) from that of the year 2017 for these regions. Though the *bio10* turned out to be the most influential predictor overall, however, its values were taken from the preceding year in the model. That said, the influence of quarterly features from the year 2018 then becomes critical to the 2018 outbreak for these regions. The observed temperatures for the 2^nd^ quarter (*max_temp_02, mean_temp_02*) were ranked as the second and fourth most important predictors by SHAP. It was found to be significantly (p<0.05) higher (>1 °C) for 2018 compared to the preceding year. On the other hand, the NDWI of the first two quarters of 2018 (Figure 4, A) were not differentiating pinpointing the pivotal role of high ambient spring temperatures in the outbreak for these regions.

## Discussion

### The rationale for using AI in predicting WNV-outbreaks

Understanding the complex interplay between eco-climatic drivers and other factors associated with WNV transmission is intricate, particularly at a high spatio-temporal scale. Novel computing methods, accounting for these complex processes in order to understand WNV-outbreak risk have rarely been applied at the European level. To this end, we attempted to bridge this gap by identifying the most influential drivers retrospectively.

To the best of our knowledge, this is the first study that combines AI and XAI frameworks to assess WNV-outbreak risk, using a time series (2010-2019) of disparate predictors, at a high spatial resolution for Europe. The XGBoost algorithm is being used widely in diverse fields of natural science and biomedical research, due to its computational power and ability to optimize model performance through parameter tuning.^70^, ^71^, ^72^ Some recent instances of the applications of the gradient boosting algorithms related to the WNV risk predictions have been reported.^49^^,^^68^ In this study, we used XGBoost to classify the NUTS3 regions with and without WNV transmission using the human WNV infections data from 2010 to 2019. Importantly, the model showed a robust predictive ability (AUC scores 0.97 and 0.93, respectively for out-sample data) of the entire WNV transmission season ahead of time. These results were also post-processed, using a game-theoretic XAI framework, SHAP, in addition to the algorithm's internal evaluation metrics, to determine the feature importance and contribution.^66^^,^^67^ Detailed discussion on the preferential selection of SHAP over XGBoost's internal metrics can be found in the appendix (see section XGBoost internal metrics versus SHAP).

### Eco-climatic factors: the key drivers of the WNV transmission

The mean temperature of the warmest quarter of the preceding year was the most important driver of WNV-outbreak European-wide. This becomes critical as a vast majority of WNV-outbreaks previously peaked during July-September.^13^ Our predictions highlight an important aspect; the current year's mean summer temperature could well indicate a WNV-outbreak in the coming year. The key aspect is that the mean temperature of the warmest quarter ranged between 20-26°C in regions with WNV presence (Appendix; Fig. A4). A similar temperature range was mechanistically shown to be the most suitable for the increased WNV incidence risk validating our findings.^39^ Likewise, a higher than usual spring temperature in this range (22-26°C) (Appendix; Fig. A4) early in a year could also be a precursor of WNV-outbreak during the latter half of the same year. The climate-related predictions of our model are in agreement with previously published studies in a similar context.^16^ Indeed, the evidence of a positive relationship between higher WNV transmission risk and temperature has been previously reported.^24^^,^^26^^,^^27^^,^^29^^,^^73^^,^^74^

The influence of higher temperature is found to increase the replication rates of WNV and its vectors.^42^ This mechanism then forces the vectors to transmit WNV earlier by shortening the gonotrophic cycle, resulting in an increased biting rate.^75^ Furthermore, due to global warming, there is an increasing trend of extreme weather events such as heatwaves, floods, or droughts. These events intensify the interaction between disease hosts, vectors, and viruses that results in favoring the transmission of a virus to humans.^76^^,^^77^

Our results also highlight that the winter climate conditions can also be predictors of WNV-outbreak risk. We found that the precipitation of the coldest quarter from the preceding year *(bio19)* was among the most influential features in our model (Figure 3, A). Our analysis showed that the lower precipitation was positively associated with higher WNV-outbreak risk (Figure 3, A). However, conflicting evidence of a strong association between the preceding year's precipitation and human WNV incidence exists.^78^ In fact, a strong negative association between annual rainfall of the preceding year and the regional level human WNV incidence risk was reported.^77^ In another study,^25^ a positive association was found between WNV presence and total days with precipitation in late winter-spring. Ecological studies suggest that the drought events can lead to outbreaks the following year due to changes in the mosquito food web structure.^79^ Mechanistic studies aiming to determine the vector ecology in varying climate conditions may well elaborate on such associations.

The NDWI, an index of water in the ecosystem, was also associated with the WNV presence (Figure 3, A). The NDWI is used as a proxy of water availability in a region. Our observed data showed a lower NDWI index during the first half of the year for all regions. Evidence of anomalies between lower NDWI and WNV risk in Europe was also reported previously.^25^^,^^26^ Lower precipitation in winter implies less water availability in the region, indicative of drought-like conditions. Such conditions are a likely indicator of the aggregation of host birds and the vectors at available water bodies, which could amplify the virus transmission rates,^78^ and influence the vector competence.^80^

### Role of host vectors in the WNV-outbreaks

The *Culex modestus*, the winter diapausing adult species,^81^ was also ranked as an influential feature by SHAP, showing a positive association between its abundance and WNV-outbreaks (Figure 3, A). The WNV affected regions had a higher vector abundance compared to those without WNV presence. Similar conclusions were drawn in other studies too.^31^^,^^33^^,^^82^ The abundance *of Culex pipiens* was also found to be higher in 2018. However, our model predicted that the regions with abundant *Culex modestus* were at more WNV-outbreak risk.

Analogous to our regional-level predictions (Appendix; Fig. A5), concurrent risk indicators were positively associated with the WNV vector abundance in other regional-level studies.^16^^,^^82^ In a regional study of the 2018-outbreak in Italy,^16^ the 2018 spring temperature was compared with the previous year's WNV-outbreak indicating an anomaly that could have played a role in amplified WNV transmission at the beginning of the season.

### WNV epidemiology and the need for cross-disciplinary public health practice

An after-action review was conducted by ECDC after the 2018 WNV outbreak that demonstrated the benefits of cross-sectoral and cross-disciplinary approaches to preparedness for WNV outbreaks in Europe.^83^ Precautions were recommended to foster and strengthen arrangements that enable coordinated One Health surveillance and response during WNV transmission seasons; ensure adequate laboratory capacities; strengthen risk communication, and fund longer-term research to address the knowledge gaps identified in this after-action review.

Our study can help address some of the knowledge gaps identified in that after-action review in order to improve the response to WNV transmission in Europe. It can help tailor prevention and response activities geographically and temporally. Our study was data-driven, and we did not account for mechanistic processes of these WNV drivers, though one could also consider those to analyze or explore the WNV-outbreak dynamics. While both the modelling paradigms uniquely address the epidemiological hypothesis of interest, a combination of both can help understand the intricate processes driving the WNV transmission. This, in essence, can help make more realistic and evidence-based largescale spatio-temporal WNV predictions and guide the public health response.

### Roadmap to AI-based WNV early warning system

WNV outbreaks can have far-reaching implications, not only for individuals but also for society at large, including the tourism industry. Contamination of blood banks by donors infected with WNV also represents a significant threat to the blood supply.^84^ Early detection of WNV outbreaks can accelerate the public health response, and reduce the risk to individuals, the economy, and blood banks. Thus, building a reliable EWS for WNV remains a public health priority.

However, the complexity of WNV transmission has proven to be challenging at best. A number of attempts have been made with entomological surveillance by focusing on interventions that enable the early detection of virus circulation in mosquitoes. These models require data on mosquito populations and the environment. Such EWS rely on data from field investigations which is hard to come by on a timely basis. Therefore, these efforts have not yielded operationalized EWS on a European scale. In contrast, our model does not require time-sensitive entomological input data from the field and can therefore be operationalized by public health authorities. Further, it can be deployed at a finer spatial resolution (NUTS3 level) with a season's lead time as well.

There are several steps involved to build an efficient and reliable EWS for a disease with the complexity of WNV. They include determining the influence of weather and other environmental variables on disease ecology to the surveillance and screening and engaging the decision-makers with risk forecasts to intervention strategies. ^85^

The EWS built as such must be evaluated and refined continuously by addressing the underlying challenges and minimizing their limitations. This can be done by following the guidelines from the recent literature on the WNV roadmap to the EWS.^86^ Further, strategies adopted to build the EWS for other diseases can also be utilized for this purpose.^87^

In this Europe-level spatio-temporal WNV-outbreaks study, we found that the seasonal climate patterns, the environmental factors, and the WNV vectors abundance were crucial characters of the past WNV-outbreaks. The rising global climate change impact in recent years indicates a further geographic expansion of WNV to previously naïve European regions. To this end, our results suggest that a climate-related WNV early-warning system that can account for and explain the intricate interplays between climatic and other drivers is necessary to achieve the European Climate and Health Observatory goals. The presented model can serve this purpose. This can be done, for example, by using future climate data to identify, inform and prepare for the potential spatial hotspots for WNV transmission in Europe in the near future. Finally, similar indicators can be developed to forecast other infectious diseases at the European scale.

## Declaration of interests

All authors declare no competing interests.

## Appendix Supplementary materials

Image, application 1
