# Supplementary material for: Artificial intelligence to predict West Nile virus outbreaks with eco-climatic drivers
Source: Lancet Reg Health Eur. 2022 Mar 30;17:100370. doi: 10.1016/j.lanepe.2022.100370 (PMC8971633; doi:10.1016/j.lanepe.2022.100370)
Supplement: Supplementary file 1 [file mmc1.docx]

**Appendix**

Artificial intelligence to predict West Nile virus outbreaks with eco-climatic drivers

Zia Farooq, Joacim Rocklöv, Jonas Wallin, Najmeh Abiri, Maquines Odhiambo Sewe, Henrik Sjödin, Jan C. Semenza

**Spatio-temporal data selection process**

The feature selection procedure was based on WNV related literature review. We searched PubMed, Web of Science, and Google scholar for primary research studies published between January 2000 and August 2021. We used combinations of "West Nile", "West Nile virus" in the title. We then filtered the European continental and regional studies. This set of research publications was furthermore filtered. Search terms for this secondary filter included: "risk", "model", "predictor", "estimate", "determinant", "driver" “machine learning” “artificial intelligence”. We extracted and complied with the determinants presented. We then stratified the list into eight categories based on class and source of data.

There on, for each feature class, we curated a range of features. Climate, environmental, demographic, and vectors distributions classes consisted of multiple features. We left out some features that were otherwise mentioned to be important in literature during the curation process. Some of these include Palmer Drought Severity Index (PDSI), a drought-condition indicator. We hypothesized that similar information could be extracted from the precipitation-related quarterly features. In addition, indices for interdecadal climatic systems such as the “El Nino South Oscillation” (ENSO) and the “North Atlantic Oscillation” (NAO) were left out in the main features classes. Other regional level factors like wetlands and land use were also not considered in this study either due to the non-availability of data or at regional level analysis previously.^1,2^

**Climatic, bioclimatic, and environmental features**

All climate-related features were extracted from the C3S database at the high spatial resolution of 0.5 x 0.5-kilometer grid.^3^ That was done for each individual NUTS3 region of European countries. Geometries of each NUTS3 region were obtained using the *Eurostats* package in R.^4^ In ecological niche modelling, the “bioclimatic” features are a commonly used set of time-varying geo-climatic metrics.^5^ Out of 19 bioclimatic features, 11 were derived from standard temperature variables, whereas, the remaining were derived from the precipitation variables for the region over time. We first extracted temperature and precipitation data spatially and temporally for the study period as mentioned above. The extracted climate data was then fed into R’s *dismo*package to extract all the bioclimatic features.^6^ The environmental indicators (NDVI, NDWI) are predictors for WNV risk at regional or national level studies and are used extensively. The NDVI data were first aggregated for each region at 0.5 x 0.5-kilometer spatial grid over time. Precisely, monthly aggregated data of each year was then averaged and transformed into four quarters. Although NDVI measures the vegetation biomass of an area, it is sensitive to spatio-temporal variability in the climate, which influences trends in disease incidence that vary across different regions.^7^ Similarly, NDWI data was derived at 0.5 x 0.5-kilometer regional scale through Google Earth Engine from 16-day Landsat-7 reflectance using the R package ‘*rgee’*.^8^ The vectors data consisting of all vectors distribution was obtained from European Center for Disease Control (ECDC).^9^ The yearly data for the regional age-structured demographic features, trading as a proxy of mobility, and economy were obtained from *R’s Eurostat* package.^4^ The host birds’ data was obtained from the European Environment Agency (EEA).^10^

The countries not included in this study are Russia, Ukraine, and Switzerland due to the unavailability of data. The WNV cases data were quality-controlled and obtained from ECDC for each NUTS3 region. Both asymptomatic and human cases with neuroinvasive WNV disease were included in the analysis. Besides the latter being more severe presenting with meningitis, encephalitis, and acute flaccid paralysis, the former constitutes the majority (about 80%) of the WNV infections.^7^ Infections with unknown etiology were excluded from the study. Figure A1 represents the yearly number of WNV cases for each country. Figure A2 shows the corresponding NUTS3 regions for each year with the scale of WNV cases.

Figure A1: All European countries with WNV cases during the study period. Fourteen WNV affected countries from southern and central Europe are shown. Year to year, the number of WNV cases per country varies significantly, but clearly, the 2018-outbreak was the intense one. Hungary, Italy, and Romania had recurring WNV cases trends followed by Greece. The bubble size represents the number of WNV cases per year per country. The bigger the bubble size, the greater the number of cases in a country per year. Overall, Greece, Italy, Hungary, and Romania had the largest number of WNV cases every year.

Figure A2: Geographic distribution of WNV cases for each year included in the study. Each subplot shows the yearly number of WNV cases of the NUTS3 regions included in this study. The maps represent only WNV affected regions of Southern and central Europe. Year to year, the number of WNV cases varies substantially geographically. From 2010 to 2019, the overall expanding geographic spread is observed.

**Machine learning algorithm and model selection**

Many data-driven algorithms were available for binary classification. From simple logistic regression and Generalized Additive Models (GAMs)**^11^** to more complex data-driven algorithms including but not limited to random forests*,***^12^** decision trees,**^13^** XGBoost, **^14^** and several others were trained and tested. This was done on a uniform data set, i.e., scaled and without any missing observations. The XGBoost algorithm outperformed the rest when applied to test data sets. The *logloss* scores and the AUC scores of the test data sets for some of the top-performing algorithms are listed in Table-A1.

As mentioned in the previous chapter, many observations in this dataset are not complete. Combining the temporal and non-temporal predictors introduces a repetition of these missing values in the produced data. To handle these missing values, one conservative choice is to discard the incomplete instances. However, we also decided to investigate another strategy to estimate missing values and evaluate the effect of the complete dataset in the classification. We used XGBoost’s sparsity-aware split finding algorithm to handle the missing values, presenting the results in the next chapter.

Though XGBoost is considered robust against highly skewed or correlated data requiring minimal data preprocessing, still, a feature level inspection helps improve model predictive power. Accordingly, the features with less than four distinct observations across training data were dropped, all belonging to Ornithological classifications. Finally, any related features to NUTS and year have been removed since the study's primary goal is to find the most influential features on the virus transmission, regardless of the observations' information on the year and NUTS identities.

Two feature classes, climatic and environmental, consisted of features with quarterly data while the rest were yearly. This, in essence, allowed us to experiment and try several explanatory models. Since all the analyses were retrospective to assess the WNV-outbreaks, we analyzed all possible scenarios to choose the best predictive model.

The model selection process was dependent on many aspects. For instance, what was the objective of estimating the predictions with multiple models? The fact that the study was retrospective, including full year’s data from periods, overlapping and beyond the WNV-outbreak seasons (usually between July-September, in Europe), was subjective, which could give drivers of both usual and intense WNV-outbreaks. We thus strategized and opted for multiple models to choose the best prospective early-warning system which could predict a WNV-outbreak ahead of time. Starting with the full year’s data, model-Q4 explained the overall key drivers retrospectively. Having the decade-long spatio-temporal data, therefore, allowed us to try the quarterly models. That said, we constructed and explored four models each. The differentiating factors between these models were the quarterly features data and yearly data features data. Features classes with quarterly data (climate, environment) were straightforward to select for each individual model. Model-Q1 contained the feature space of first-quarter features of these two classes. However, for the rest of the features, only yearly data was available. For a model to act as an early-warning system to predict outbreak risk within a year, assumptions about these features had to be made. Accordingly, spatio-temporal values for these features were taken from the preceding year’s data in each region. That is, the data of any NUTS3 region for any of these features for the year 2011 was taken from the year 2010 data and for 2012 from 2011 and so on. To avoid repetitiveness, the year 2010 was left out as it needed to have the previous year’s data which was out of the study period.

The best explanatory algorithms were selected by deploying and assessing the performance on probabilistic metric *logloss*. The XGBoost ^14^ was the standout algorithm in terms of predictive power for our data. One of the great advantages of XGBoost over other machine learning algorithms is its ability to adjust for features and require least data preprocessing and feature engineering. Moreover, it can handle well highly nonlinear and interactive covariates. For each of the four models, the final data set was divided into training/validation (2010-2017) and test (2018, 2019). To remove any autocorrelation of the features, the feature representing year and the geographic identification of a NUTS3 region was removed before training. Figure A3 shows the model-Q2 data of training and test periods used in the main analysis.

Figure A3: Training and test datasets features. Data visualization of the features from all feature classes except the birds features, included in the model-Q2 for training and test data. The horizontal axis represents the data set category (i.e., training or test) separated by colors. Except for the bird data due to visualization limitations, all the features’ data is shown.

| Model/ Algorithm | Logloss | AUC | | | |
| --- | --- | --- | --- | --- | --- |
|  | Test 2018 | | Test 2019 | Test 2018 | Test 2019 |
| Naïve Model^*^ | 0.43 | | 0.29 | - | - |
| XGBoost | **0.17** | | **0.18** | **0.97** | **0.93** |
| Random forest | 0.27 | | 0.18 | 0.95 | 0.93 |
| SVM | 0.30 | | 0.24 | 0.89 | 0.93 |
| Decision Trees | 0.28 | | 0.19 | 0.93 | 0.93 |
| LightGBM | 0.20 | | 0.19 | 0.95 | 0.91 |

Table-A1: Comparison of performance metrics of various algorithms on out-of-sample data sets.

*Naïve model refers to the model without any features whose *logloss* score was computed based on the constant probability equal to the proportion of positive classes in the respective data sets.

| Model | Parameters | | | | | | |  |
| --- | --- | --- | --- | --- | --- | --- | --- | --- |
|  | **nrounds** | **eta** | **gamma** | **min_child_weight** | **scale_pos_weight** | **lambda** | **max_depth** | **max_delta_step** |
| Q1 | 846 | 0.03 | 0.82 | 13 | 15.91 | 0.48 | 9 | 8 |
| Q2 | 357 | 0.04 | 1.58 | 10 | 15.91 | 0.73 | 7 | 9 |
| Q3 | 500 | 0.25 | 1.85 | 15 | 11.64 | 0.23 | 19 | 9 |
| Q4 | 500 | 0.36 | 1 | 9 | 16.18 | 0.4 | 30 | 9 |

Table-A2: The optimized hyperparameters set for each model obtained after random search.

**Table-A3:**  Performance metrics of all the models for three classification-threshold and the AUC score.

| **Threshold** | **Accuracy** | **Sensitivity** | **Specificity** | **Precision** | **F1. Score** | **Balanced.Accuray** | **Test.**  **Year** | **Model** | **AUC** |
| --- | --- | --- | --- | --- | --- | --- | --- | --- | --- |
| 0.1 | 0.93 | 0.73 | 0.96 | 0.78 | 0.75 | 0.84 | 2018 | Q1 | 0.97 |
| 0.2 | 0.93 | 0.64 | 0.98 | 0.85 | 0.73 | 0.81 | 2018 | Q1 | 0.97 |
| 0.5 | 0.91 | 0.47 | 0.99 | 0.91 | 0.62 | 0.73 | 2018 | Q1 | 0.97 |
| 0.1 | 0.88 | 0.69 | 0.90 | 0.41 | 0.51 | 0.80 | 2019 | Q1 | 0.93 |
| 0.2 | 0.91 | 0.65 | 0.94 | 0.51 | 0.57 | 0.79 | 2019 | Q1 | 0.93 |
| 0.5 | 0.93 | 0.50 | 0.97 | 0.63 | 0.56 | 0.74 | 2019 | Q1 | 0.93 |
| 0.1 | 0.94 | 0.86 | 0.95 | 0.76 | 0.81 | 0.91 | 2018 | Q2 | 0.97 |
| 0.2 | 0.94 | 0.77 | 0.97 | 0.83 | 0.80 | 0.87 | 2018 | Q2 | 0.97 |
| 0.5 | 0.93 | 0.64 | 0.99 | 0.92 | 0.75 | 0.81 | 2018 | Q2 | 0.97 |
| 0.1 | 0.91 | 0.69 | 0.93 | 0.49 | 0.58 | 0.81 | 2019 | Q2 | 0.93 |
| 0.2 | 0.92 | 0.56 | 0.96 | 0.57 | 0.56 | 0.76 | 2019 | Q2 | 0.93 |
| 0.5 | 0.94 | 0.46 | 0.99 | 0.80 | 0.59 | 0.72 | 2019 | Q2 | 0.93 |
| 0.1 | 0.88 | 0.85 | 0.89 | 0.58 | 0.69 | 0.87 | 2018 | Q3 | 0.93 |
| 0.2 | 0.90 | 0.77 | 0.92 | 0.65 | 0.71 | 0.85 | 2018 | Q3 | 0.93 |
| 0.5 | 0.91 | 0.62 | 0.97 | 0.79 | 0.70 | 0.80 | 2018 | Q3 | 0.93 |
| 0.1 | 0.93 | 0.67 | 0.95 | 0.60 | 0.64 | 0.81 | 2019 | Q3 | 0.94 |
| 0.2 | 0.94 | 0.58 | 0.97 | 0.68 | 0.62 | 0.77 | 2019 | Q3 | 0.94 |
| 0.5 | 0.94 | 0.40 | 0.99 | 0.84 | 0.55 | 0.70 | 2019 | Q3 | 0.94 |
| 0.1 | 0.90 | 0.75 | 0.92 | 0.65 | 0.69 | 0.84 | 2018 | Q4 | 0.92 |
| 0.2 | 0.92 | 0.70 | 0.96 | 0.75 | 0.73 | 0.83 | 2018 | Q4 | 0.92 |
| 0.5 | 0.91 | 0.55 | 0.98 | 0.83 | 0.66 | 0.76 | 2018 | Q4 | 0.92 |
| 0.1 | 0.92 | 0.67 | 0.95 | 0.56 | 0.61 | 0.81 | 2019 | Q4 | 0.95 |
| 0.2 | 0.93 | 0.58 | 0.97 | 0.64 | 0.61 | 0.77 | 2019 | Q4 | 0.95 |
| 0.5 | 0.92 | 0.31 | 0.99 | 0.70 | 0.43 | 0.65 | 2019 | Q4 | 0.95 |

XGBoost internal metrics versus SHAP

In simple classification algorithms such as logistic regression, the value of estimated coefficients indicates the importance of a feature in the model. Contrarily, XGBoost being a tree-based algorithm, ranks the feature importance based on its own internal metrics. It has three such metrics: *frequency, cover,* and *gain.^14^* The feature importance is generally based on *gain* score, though the other two can also be used. However, evidence exists that the SHAP is a more robust approach for feature importance ranking.^14^ Like any other tree-based algorithm, the algorithm makes iterative features selection that best separates the data into two groups. Thereafter, on each iteration, a feature score is assigned to select the optimal split.^15^ The conflict arises when features have an equal score during the training process at a given level. This is where the XGBoost priority is given to the first one in terms of the insertion order of the features. This is somewhat conflicting with other tree-based implementations because they would select features randomly. This is where SHAP framework work comes into play as it accounts for every possible combination and order of features. The importance of a feature is ranked from its marginal contribution to the model output. XGBoost gains score is highest for *bio10,* making it the most important feature. The order of the remaining features is slightly different compared to SHAP estimated list.

The SHAP framework is, in fact, a matrix of the dimension of data and is a robust method to reverse-engineer a predictive model’s outputs. Coupling an XGBoost like black-box model with SHAP makes these models more explainable than even simple logistic regression type models. Shapley algorithm,^16^ quantifies the individual contribution of each player (feature) to the game (outcome of one observation). This is done for each observation individually to predict every single model outcome with and without the presence of each feature. The instance-level predictions are then aggregated to quantify the overall feature importance. Figure A4 represents the actual feature value and SHAP value relationship of the top-4 features predicted by SHAP for the model-Q2. The fact that SHAP analyzes a model prediction locally allowed us to compute the instance-level feature exploration and contributions to the overall model predictions.

Figure A5:  Feature values classification and contribution estimated by SHAP. A) The mean temperature of warmest quarter *(bio10)*; B) Maximum temperature of 2^nd^ quarter (*max_temp_02*); C) Temperature seasonality (bio4); D) Mean temperature of 2^nd^ quarter. The x-axis represents the actual feature values, and the y-axis represents the corresponding SHAP value (*log-odds*). The binary color shows the class to which feature value belongs, that is, 1 for a region with WNV presence and 0, for otherwise. Feature value with a positive SHAP value is interpreted to have contributed or pushed the model towards the class of interest, i.e., the WNV presence in a region, whereas a negative SHAP value flips the role of the feature in the instance prediction.

Regional-level key WNV drivers predicted by SHAP?

Every NUTS3 region represents one data point. Thus, we explored how the model yielded a prediction for a single NUTS3 region using SHAP. We selected a set of NUTS3 regions that exceeded a threshold of 50 WNV cases in 2018 to explore their local drivers (Figure A5). Four regions were found to fulfill this criterion, three from Italy and one from Romania. While overall preceding year's climate trends were key predictors, small regional-level variabilities were observable. For all Italian regions, *bio10* was the key predictor of the 2018 WNV-outbreak, like the European-wide prediction. However, the regional predictions for the Romanian region differed, and bio*17* was found to be the top predictor followed by *bio10*. A regional cross-analysis revealed differences in the relative importance of the predictors. For instance, the abundance of *Culex modestus* vectors was ranked more critical for Padova and Bologna than the European-wide predictions (Figure 5). Similarly, the past year's trade-related mobility features were among the top drivers of WNV-outbreak for these two regions but not the other regions examined. In contrast, early year's NDVI index *(ndvi_01)*for Modena and Bucuresti regions were among the key predictors of WNV-outbreaks but not for the two other regions.

Figure A5) Regional-level SHAP predictions: Regions with the greatest number of WNV cases during the 2018-outbreak are shown. The regional-level absolute SHAP values along the x-axis are shown. The features are ranked according to their importance along the y-axis. The model correctly classified all these regions as WNV positive with a standard classification threshold of 0.5.

Figure A6: Model-Q2: XGBoost’s internal metrics (frequency, gain, cover) plot. The feature importance from XGBoost all three metrics. Frequency: the percentage of the relative number of times a feature has been used in trees, Cover: metric of the total number of observations related to this feature, Gain: the fractional contribution of each feature to the model based on the total gain of this feature's splits. The importance of a feature is related to its position in the plane, i.e., more important features with respect to all three metrics will be the ones having bigger bubble size and are placed upper-right of the plane. *bio10*was the most pronounced feature ranked by XGBoost metrics as well. However, the order of ranking for other features differs from the SHAP estimated rankings.

Top drivers predicted by model-Q1, model-Q3, and model-Q4:

Below, the top predictors for each of the model-Q1 (Figure A7), model-Q3 (Figure A8), and model-Q4 (Figure A9) are shown. The model-Q1 had the smallest feature space while model-Q4 consisted of the biggest feature space. Model-Q4 differs from the others in that no feature values are taken from the preceding year's estimates. Overall, the key drivers remain similar for all the models, with slight variations in rankings.

Figure A7: Model-Q1: Top-10 most important SHAP predicted features. The top contributing features are the preceding year’s *bio10*, the mean temperature of the warmest quarter from the preceding year, the temperature seasonality *(bio4),*the NDWI index of the first quarter of the same year (*mndwi_q1),*and distribution of the Culex modestus vectors (*dist_Culex.modestus*) from the preceding year.

Figure A8: Model-Q3: Top-10 most important SHAP predicted features. The top contributing feature is the *mean_temp_02*, the mean temperature of the 2nd quarter of the year, followed by bio10, the mean temperature of the warmest quarter of the preceding year, *min_temp_02,* the minimum temperature of the 2nd quarter of the year*,*and the temperature seasonality *(bio4)*. Other than temperature, the precipitation of coldest quarter (*bio19*), the NDWI of 2nd quarter (*mndwi_q2*), and the NDVI of the first quarter (*ndvi_01*) of the year were also amongst the top predictors.

Figure A9: Model-Q4: Top-10 most important SHAP predicted features. The top contributing feature is the *bio10*, the mean temperature of the warmest quarter of the year, the temperature seasonality *(bio4), and the mean_temp_02,* the mean temperature of the 2nd quarter of the year. The model-Q4 was the full year’s data model and does not contain feature values from the preceding year’s estimates.

Imputation of the incomplete dataset using XGBoost

The original incomplete data contain 13420 observations with most of the incomplete observations from the regions that had no virus presence or transmission reported during the study period. Our final data sets for the models Q1-Q3 and model-Q4 contained 5048 and 5609 complete observations, respectively. The class-wise proportion of positive/negative observations was approximately 7% / 93% in all the data sets showing a high class-imbalance. In the main text and previous sections, we used these data sets with complete observations to handle the missing values. We did so since we found that the results were sensitive to the imputation of some features classes, particularly from the eco-climatic and vectors. Nevertheless, we still had a good amount of data for our classification algorithm to do the predictions since most of the incomplete observations were from the regions that had no virus presence or transmission during the study period.

Besides removing incomplete observations, there are other ways to deal with missing values, such as setting them to zero and several imputation methods.^17,18^ XGBoost proposed a sparsity-aware split finding algorithm that learns the optimal default direction by only visiting the non-missing entries.^14^

Before blindly using the imputation, we realize that estimating several features for each NUTS3 region can be achieved by looking at the NUTS3 observed neighbors. By looking at each NUTS3 with missing values at the climate and bioclimatic features, we measure the average over the observed ones to estimate them. For demographic attributes of a NUTS3 region, the values of a missing year are replaced by the nearest observed years.

The remaining missing features are the three groups of host birds, vectors, and socio-economic, which will be estimated using XGBoost during training the classifier. The missing values in the dataset that is sent to XGBoost are shown in Figure A10. As seen in the figure, an overwhelming majority of the features consist of bird types. However, only a minimal number of them are observed. The missingness pattern seen for these features is because they are not collected yearly, and we had to repeat them regarding their spatial locations.

**
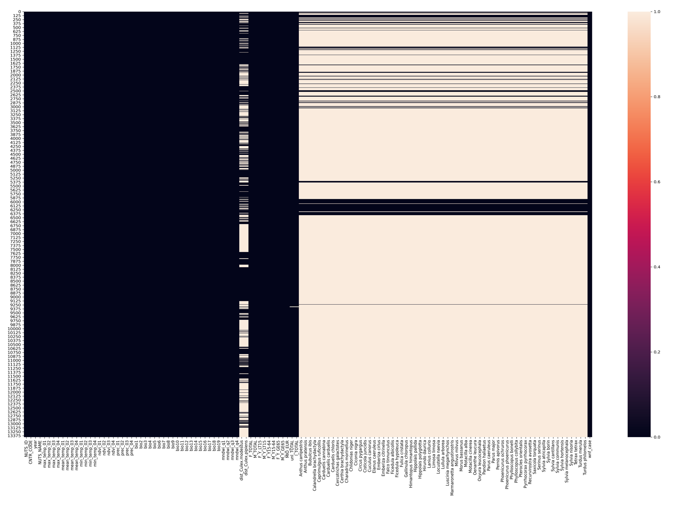
**

Figure A10: Missing Trends in the data: White shows the missing values, and black indicates the observed ones. The missing values in climate, bioclimatic and demographic features are estimated by their NUTS3 neighbors. Two vectors features, socio-economics, and sixty-two birds features were imputed using XGBoost.

After training the XGBoost classifier with data up to 2018, we used the SHAP to show the importance of the features in Figure A11.


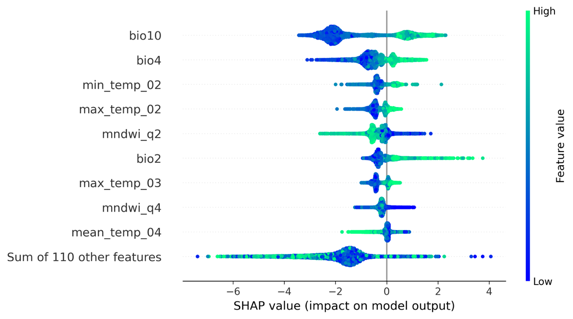


Figure A11: The top contributing features predicted by SHAP for imputed dataset using XGBoost.

Further, we test the trained classifier on both the 2018 and 2019 datasets. Like the previous section, the results for accuracy, F1 score, and other metrics are shown in table A4.

| Thresholds | 0.1 | | 0.2 | | 0.5 | |
| --- | --- | --- | --- | --- | --- | --- |
| Test years | 2018 | 2019 | 2018 | 2019 | 2018 | 2019 |
| Accuracy | 0.87 | 0.94 | 0.91 | 0.95 | 0.93 | 0.96 |
| Sensitivity | 0.74 | 0.66 | 0.61 | 0.54 | 0.46 | 0.41 |
| Specificity | 0.88 | 0.96 | 0.94 | 0.97 | 0.98 | 0.99 |
| Precision | 0.42 | 0.48 | 0.52 | 0.55 | 0.70 | 0.67 |
| F1 score | 0.53 | 0.55 | 0.57 | 0.54 | 0.56 | 0.51 |

Table A4: results for three different classification thresholds. The results are for two different test data from 2018 and 2019.

In table A5, we trained the model up to 2019 (the year 2018 is part of the training data) and tested the model for 2019. The important features are shown Figure A12.

| Thresholds | 0.1 | 0.2 | 0.5 |
| --- | --- | --- | --- |
| Test years | 2019 | 2019 | 2019 |
| Accuracy | 0.89 | 0.92 | 0.96 |
| Sensitivity | 0.89 | 0.83 | 0.73 |
| Specificity | 0.89 | 0.93 | 0.97 |
| Precision | 0.32 | 0.40 | 0.61 |
| F1 score | 0.47 | 0.53 | 0.67 |

Table A5: results for three different classification thresholds. The results are for 2019 test data while the model is trained for the previous years.

**
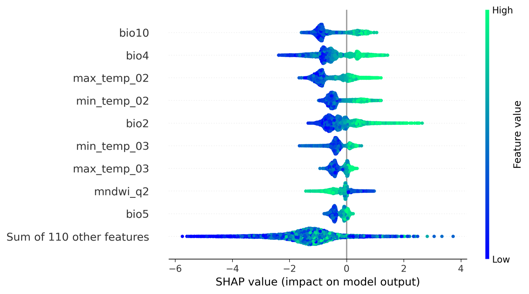
**

Figure A12: The nine top contributing features using the SHAP. The model has been trained on all years up to 2019, which is used for testing.

The alternative strategy for birds features

As mentioned, most bird types’ features are missing, which was previously handled with XGBoost. Additionally, we also consider the case that these values are missing since they haven’t been seen to be reported (no news is zero news). Setting all of the missing bird values to zero, we repeated the previous analysis.


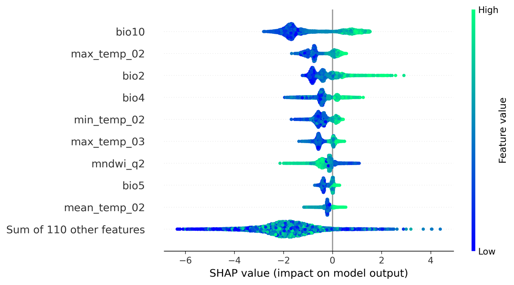


Figure A13: The nine top contributing features using the SHAP. All the missing values of bird features are set to zero.

| Thresholds | 0.1 | | 0.2 | | 0.5 | |
| --- | --- | --- | --- | --- | --- | --- |
| Test years | 2018 | 2019 | 2018 | 2019 | 2018 | 2019 |
| Accuracy | 0.83 | 0.93 | 0.87 | 0.94 | 0.93 | 0.96 |
| Sensitivity | 0.85 | 0.78 | 0.78 | 0.67 | 0.62 | 0.51 |
| Specificity | 0.82 | 0.93 | 0.89 | 0.96 | 0.96 | 0.98 |
| Precision | 0.35 | 0.4 | 0.44 | 0.47 | 0.65 | 0.64 |
| F1 score | 0.5 | 0.53 | 0.56 | 0.55 | 0.63 | 0.57 |

Table A6: results for three different classification thresholds. The results are for two different test data from 2018 and 2019.

| Thresholds | 0.1 | 0.2 | 0.5 |
| --- | --- | --- | --- |
| Test years | 2019 | 2019 | 2019 |
| Accuracy | 0.93 | 0.94 | 0.95 |
| Sensitivity | 0.71 | 0.64 | 0.51 |
| Specificity | 0.94 | 0.96 | 0.98 |
| Precision | 0.40 | 0.48 | 0.55 |
| F1 score | 0.52 | 0.55 | 0.53 |

Table A7: results for three different classification thresholds. The results are for 2019 test data while the model is trained for the previous years.

**
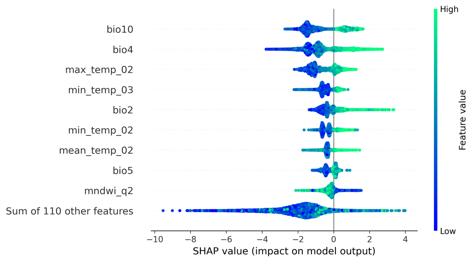
**

Figure A15: The nine top contributing features using SHAP using data. All the missing values of bird features are set to zero. The model has been trained on all years up to 2019, which is used for testing

Further discussions

Although the incomplete dataset is extremely imbalanced in favor of the negative class, i.e., regions without WNV cases, XGBoost with sparsity-aware split finding algorithm was able to find the reasonable direction on the trees for the missing values and classify the test datasets with high accuracy.

While the *Culex pipiens* and *modestus* vectors are associated with outbreak risk, other mosquitos like *Culex restuans*, *Quinquefasciatus*, *Mansonia richiardii* have also been found important in this context.^19,20^ The spatio-temporal data was not available except for the first two and were not included in the study. Although their inclusion might not have impacted overall model predictive power, regional risk indicators could have been influenced. Similarly, the WNV host birds are pivotal in the disease spread and are considered as the host reservoirs of the virus. The orders of *passeriform*, *gruiformes*, *columbiformes* and *pelecaniformes* are associated with WNV risk. Here only spatial data was available for the *passeriform* order birds at a limited scale. Unfortunately, the European level spatio-temporal birds’ data for most of the regions were not present and documented for the period of study, a drawback we wished to circumvent outwardly. Though a more complete birds data at the NUTS3 level could impact the model's feature importance, we analyzed that the model’s predictive power without birds features remained like the full feature space. Despite these shortcomings, the presented modelling framework considers the fine-scale geospatial data necessary to mimic the highly complex and intertwined processes involved in the dissemination of a WNV-like infectious disease. Evidently, in all our analyses eco-climatic were the most pronounced predictors of WNV transmission for any region.

Coupled with state-of-the-art data-driven frameworks like XGBoost and advanced explanatory algorithms like SHAP, arguably it was the most appropriate set of tools to analyze a problem of this complexity at such a large scale.

Reference:

1. Rosà R, Marini G, Bolzoni L, et al. Early warning of West Nile virus mosquito vector: climate and land use models successfully explain phenology and abundance of Culex pipiens mosquitoes in north-western Italy. *Parasites & Vectors* 2014; 7(1): 1-12.

2. Marcantonio M, Rizzoli A, Metz M, et al. Identifying the environmental conditions favouring West Nile virus outbreaks in Europe. *PLoS One* 2015; 10(3): e0121158.

3. Copernicus Climate Change Service  https://climate.copernicus.eu/the-climate-data-store (accessed November 16 2021).

4. Lahti L, Huovari J, Kainu M, Biecek P. Retrieval and Analysis of Eurostat Open Data with the eurostat Package. *R J* 2017; 9(1): 385.

5. Kriticos DJ, Jarošik V, Ota N. Extending the suite of bioclim variables: a proposed registry system and case study using principal components analysis. *Methods in Ecology and Evolution* 2014; 5(9): 956-60.

6. Hijmans R, Phillips S, Elith J. J (2017) dismo: Species Distribution Modeling. R package version 1.1-4.

7. Liu Y, Li Y, Li S, Motesharrei S. Spatial and temporal patterns of global NDVI trends: correlations with climate and human factors. *Remote Sensing* 2015; 7(10): 13233-50.

8. Aybar C, Wu Q, Bautista L, Yali R, Barja A. rgee: An R package for interacting with Google Earth Engine. *J Open Source Softw* 2020; 5: 2272.

9. European Center for Disease Prevention and Control. https://www.ecdc.europa.eu/en. (accessed November 16 2021).

10. European Environment Agency. https://www.eea.europa.eu/. (accessed November 2021).

11. Wood SN. Stable and efficient multiple smoothing parameter estimation for generalized additive models. *Journal of the American Statistical Association* 2004; 99(467): 673-86.

12. Ho TK. Random decision forests. Proceedings of 3rd international conference on document analysis and recognition; 1995: IEEE; 1995. p. 278-82.

13. Wu X, Kumar V, Quinlan JR, et al. Top 10 algorithms in data mining. *Knowledge and information systems* 2008; 14(1): 1-37.

14. Chen T, Guestrin C. Xgboost: A scalable tree boosting system. Proceedings of the 22nd ACM SIGKDD international conference on knowledge discovery and data mining; 2016; 2016. p. 785-94.

15. XGBoost Documentation. https://xgboost.readthedocs.io/en/latest/tutorials/model.html.

16. Shapley LS. Notes on the n-Person Game—II: The Value of an n-Person Game.(1951). 1951.

17. Horton NJ, Kleinman KP. Much ado about nothing: A comparison of missing data methods and software to fit incomplete data regression models. *The American Statistician* 2007; 61(1): 79-90.

18. Van Buuren S, Groothuis-Oudshoorn K. mice: Multivariate imputation by chained equations in R. *Journal of Statistical Software* 2011; 45(1): 1-67.

19. Paz S, Semenza JC. Environmental drivers of West Nile fever epidemiology in Europe and Western Asia—a review. *International Journal of Environmental Research and Public Health* 2013; 10(8): 3543-62.

20. Deichmeister JM, Telang A. Abundance of West Nile virus mosquito vectors in relation to climate and landscape variables. *Journal of Vector Ecology* 2011; 36(1): 75-85.
